# Genome-wide linkage analysis of the tracking of systolic blood pressure using a mixed model

**DOI:** 10.1186/1471-2156-4-S1-S88

**Published:** 2003-12-31

**Authors:** Tao Wang, Guohua Zhu, Kevin J Keen

**Affiliations:** 1 Department of Epidemiology and Biostatistics, Rammelkamp Center for Education and Research, MethoHealth Medical Center, 2500 MetroHealth Drive, Case Western Reserve University, Cleveland, Ohio 44109 USA

## Abstract

**Background:**

Elevated blood pressure in middle age is a major risk factor for subsequent cardiovascular complications. An important longitudinal characteristic of blood pressure is the "tracking phenomenon". Tracking is defined as the persistence of the rank of a person's blood pressure level in a group over a long period of time. In this analysis, we used the Framingham data to investigate whether there are some genes responsible for this phenomenon.

**Results:**

Both two-point and multipoint linkage analyses were applied to family members with complete data only and to all family data with missing values imputed by a Gaussian model. The results of two-point linkage analysis indicated that two loci for linkage with the intercept were on chromosomes 10 and 13, and two loci for linkage with both slope and intercept were on chromosomes 1 and 3. Multipoint linkage analysis indicated only one region, 200–240 cM on chromosome 1, to be linked with both intercept and slope. For the intercept of SBP, the highest LOD (4.43) was found at 214 cM when missing data were imputed, and the highest LOD (2.81) was at 231 cM for the complete case data. For the slope of SBP, the highest multipoint LODs were 3.63 at 227 cM and 2.02 at 234 cM for the complete case data and imputation data, respectively.

**Conclusion:**

One or more genes in the range of 200–240 cM on chromosome 1 may be related to the tracking phenomenon of SBP.

## Background

Elevated blood pressure in middle age is a major risk factor for subsequent cardiovascular complications, such as congestive heart failure, left ventricular hypertrophy, stroke, renal dysfunction, and renal failure. An important longitudinal characteristic of blood pressure is the "tracking phenomenon". Here, tracking is defined as the persistence of the rank of a person's blood pressure level in a group over a long period of time. A large number of longitudinal studies have provided evidence of "tracking" of systolic blood pressure (SBP) [1]. The tracking of SBP implies that a person with higher initial blood pressure in early life is more likely to have a larger blood pressure increase in later life. Some genes might be responsible for this tendency.

Several genome-wide scan studies in which cross-sectional SBP was used as the phenotype have been reported in the literature. The Framingham Heart Study, which began in 1948 and characterized SBP in a two-generation cohort, provides a unique opportunity to detect risk factors of SBP via longitudinal traits. In this study, a two-stage approach was used to detect genes related to the tracking of SBP. First, two phenotypes, intercept and slope, were derived using a mixed model to represent the initial value and the rate of increase in SBP. Second, both two-point and multipoint linkage analyses were conducted using these phenotypes.

## Methods

### Phenotypes

A mixed model was fitted to both the original and offspring cohorts as follows:

*Y*_*i *_= *X*_*i *_β + *Z*_*i*_*b*_*i *_+ ε_(1)*i *_+ ε_(2)*i*_

*b*_*i *_~ *N *(0, *D*)

ε_(1)*i *_~ *N *(0, σ^2^*I*_*ni*_)     (1)

ε_(2)*i *_~ *N *(0, τ^2^*H*_*i*_)

*b*_1_,...,*b*_*N*_,ε_(1)1_,...,ε_(1)*N*_, ε_(2)1_,...,ε_(2)*N*_ independent,

where *Y*_*i *_is the vector of *n*_*i *_SBP measurements (*Y*_1*i*_,*Y*_2*i*_,...,*Y*_*ni*_) for the *i*^th ^individual, each element of this vector being one observation in a time sequence for the individual *i*:*X*_*i *_and *Z*_*i *_are matrices of covariates; β is a parameter vector representing fixed effects; *b*_*i *_is a parameter vector representing random effects; ε_(1)*i *_is an extra component of measurement error, and ε_(2)*i *_is a component of error due to serial correlations.

In this analysis, all individuals having at least three SBP measurements were selected from the 330 families in the Framingham data. There were 15,509 observations from the original cohort and 6155 observations from the offspring cohort. Because individuals in the different cohorts might be different in many respects, the model was fitted to the original cohort and offspring cohorts, respectively.

Assuming that each individual profile could be approximated by a linear function over age with subject-specific intercept as well as slope, our final model is:

Y_*ij *_= β_1_M + β_2_F + β_3_BMI + (β_4_M + β_5_F + β_6_BMI) age + b_0i _+ b_1i _age + ε_(1)i _+ ε_(2)i_,     (2)

where *Y*_*ij *_is the *j*^th ^observation of individual *i*, M is a binary variable for male, F is a binary variable for female, *b*_0*i *_is the intercept, and *b*_1*i *_is the slope. Thus *b*_0*i *_and *b*_1*i *_were defined as intermediate quantitative phenotypes for individual *i*. PROC MIXED in SAS^® ^was used to fit this model assuming an unstructured covariance *D *and a Gaussian serial correlation *H*.

### Missing values and imputation

Because of the various treatments and different drug responses in patients, we decided not to use any blood pressure values recorded after the subject started to receive treatment. In this analysis, observations after the start of hypertension treatment were coded as missing.

The mixed model was fitted to both the complete case data, i.e., only individuals without missing values, and the data that included both complete and imputed cases. Because of missing values, 8988 observations in the original cohort and 41 observations in the offspring cohort were excluded in the complete case data. Imputation was based on the Gaussian model for quantitative variables, which was fitted by the EM algorithm [2] implemented in the MISSING library in S-PLUS^®^. The process was conducted separately for the original and the offspring cohorts.

### Genetic analysis

Mendelian inconsistencies and the relationships were checked using the S.A.G.E. programs MARKERINFO and RELTEST. Heritability of the intercept and slope were estimated using SOLAR.

Two-point and multipoint linkage analyses were conducted using a model-free approach. The SIBPAL program in S.A.G.E. was used for two-point linkage analysis. This program modeled the mean corrected cross product trait data from full sib pairs as a function of marker allele sharing identical by descent (IBD) [3]. The multipoint linkage analysis was performed using SOLAR, which is based on a variance-component model.

## Results

**Figure 1:**
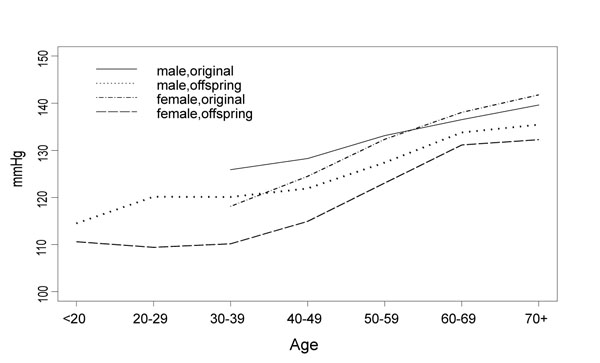
Sex- and age-specific mean of systolic blood pressure

In both cohorts, the mean SBP increases with age for both males and females (Figure 1). The mean SBP is higher in the original cohort than in the offspring cohort across ages. In the original cohort, the mean slope of SBP for females is slightly higher than for males.

To verify the tracking of SBP in the Framingham population, Pearson correlation coefficients among the different age groups were evaluated. All the correlation coefficients were statistically significant (Table 1).

The heritability of SBP was 0.321(95% CI, 0.192–0.450, *p *< 0.0001) and 0.406 (95% CI, 0.273–0.540, *p *< 0.0001) for the intercept and slope, respectively.

**Figure 2:**
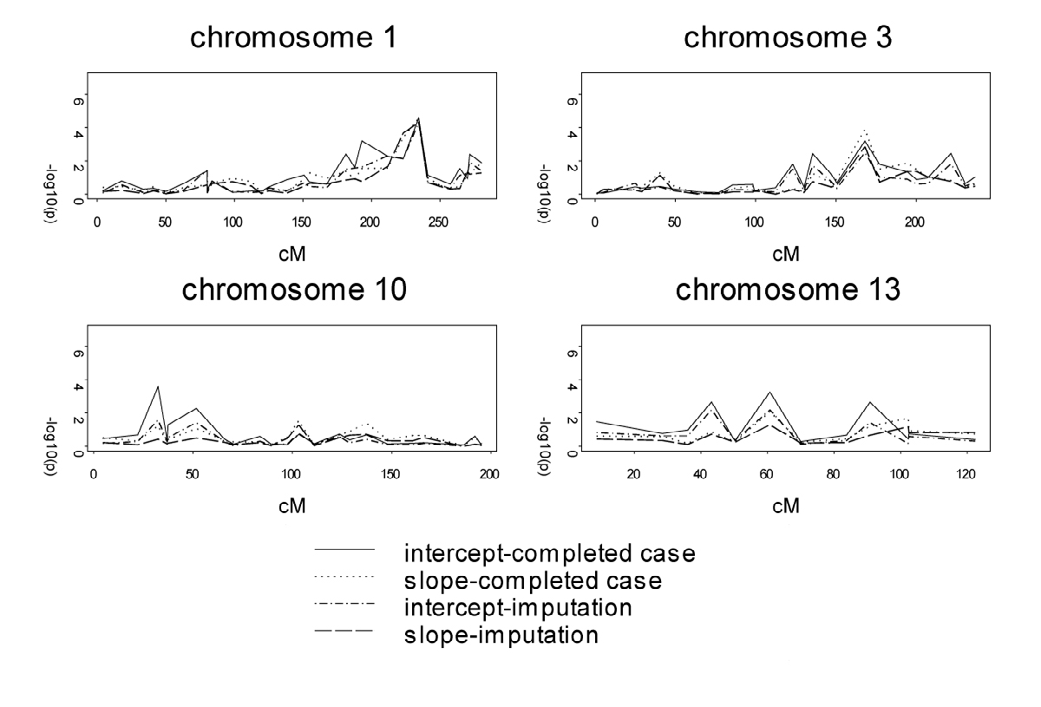
Results of two-point linkage analysis

The results of two-point linkage analysis showed two loci with suggestive evidence for linkage with the intercept on chromosome 10 (*p *< 0.001) and chromosome 13 (*p *< 0.001). The most interesting result was that linkage with both the slope and intercept was detected on chromosome 1 and on chromosome 3 (*p *< 0.0001 and *p *< 0.001, respectively) (Figure 2).

**Figure 3:**
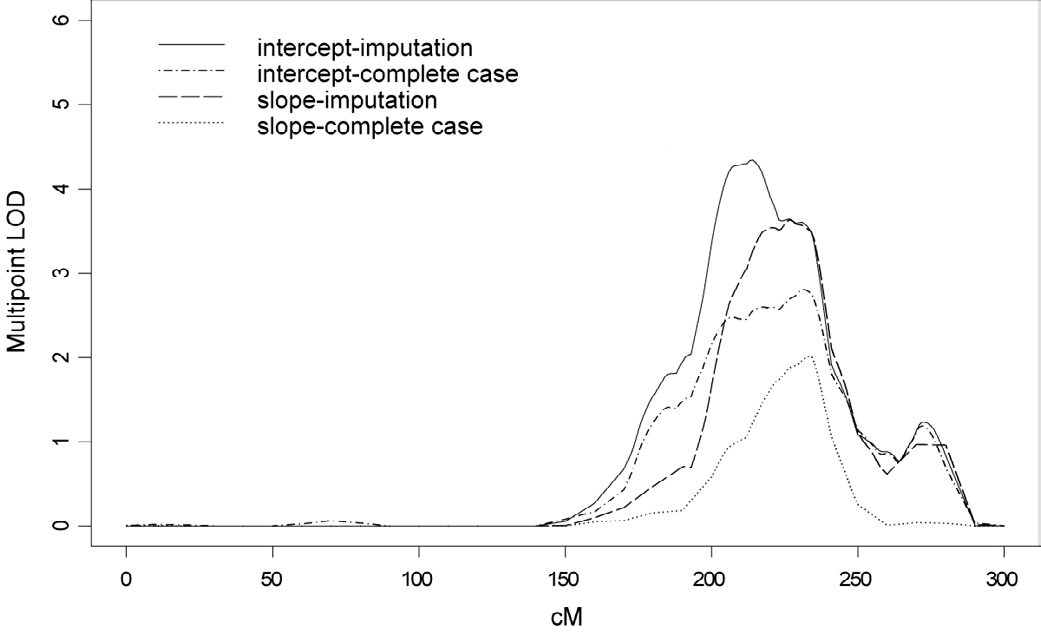
Multipoint linkage analysis: chromosome 1

Multipoint linkage analysis also indicated that the region on chromosome 1 was linked with both the intercept and slope. Figure 3 shows the multipoint LOD scores for chromosome 1. For the intercept of SBP, the highest LOD (4.43) was found at 214 cM for the imputation data, and the highest LOD (2.81) was found at 231 cM for the complete case data. For the slope of SBP, the highest multipoint LODs were 3.63 at 227 cM and 2.02 at 234 cM, for complete case data and imputed data, respectively.

## Discussion

Hypertension has been examined in various linkage studies. In those studies, the trait was either blood pressure (a continuous trait) or hypertension (a dichotomous trait). Because blood pressure changes with time, a trait which can include longitudinal information is better suited to linkage analysis than a trait that only incorporates cross-sectional information.

In order to derive this kind of intermediate phenotype for SBP, we noticed that the "tracking phenomenon" is a longitudinal characteristic of SBP. There is general agreement that tracking exists among different populations. In our analysis, all Pearson correlation coefficients were statistically significant among the different age groups, suggesting tracking also exists in our study population.

This phenomenon involves the initial value as well as the change of SBP over time, so both the intercept and slope in the random effect components of a linear mixed model were used. Because the mixed model was subject-specific, it was able to characterize individual behavior through the best linear unbiased predictor. In addition, this model made use of the prior information that phenotypes are correlated between time points, so it was considered more efficient than a standard regression model.

We hypothesized that genes play a role in the "tracking phenomenon". To test this hypothesis, the heritability of the intercept and slope were first estimated. The heritability of each trait was statistically significant, suggesting further linkage analysis would be worthwhile. To avoid false-positive results, linkage analyses were conducted based on both the Haseman-Elston model and the variance component model.

One challenge in the analysis was how to deal with the data when subjects had received treatment. The adjustment for complex diseases like hypertension is difficult, and can even be misleading, with bias that cannot be estimated. In this analysis, SBP values after receiving treatment were considered as missing, although this approach takes the risk of losing some information.

Another challenge arose from the large number of missing values. Two methods, complete case analysis and model-based imputation analysis, were used. Complete case analysis was simple but, in theory, less efficient than the imputation analysis, which assumed a distribution for all the data together, both the missing and observed data. From the result of our multipoint linkage analysis, the imputation appears to be more efficient. However, its efficiency was not shown in the two-point linkage analysis. This implies that imputation is not always efficient in linkage analysis. An inappropriate imputation model may result in more bias than the complete case analysis. In our analysis, the results from complete case data and imputation data were consistent, implying that our imputation model did not introduce large extra bias.

Our analysis was actually a two-stage approach, in which the pedigree structure was not considered at the first stage when a mixed model was fitted. An approach that simultaneously fits the mixed model and the linkage model ought to be more efficient. To fulfill this goal, further extension of the mixed model and the imputation model are needed for longitudinal pedigree data [4].

## Conclusions

In conclusion, our results provide evidence for our hypothesis that there might be gene(s), specifically in the region of 200–240 cM on chromosome 1, related to the "tracking phenomenon" of SBP. If there is only a single causative gene in this region, then it has a pleiotropic effect on both the intercept and slope of SBP. The correlation between the intercept and slope might make contribution to the consistency, but this kind of consistency on this region was not found on other chromosomes, implying that it was unlikely to be the only cause. This result provides a focus for further study, including fine mapping based on linkage disequilibrium and the evaluation of functional consequences of genes in this region.

Our results also suggested there are two other loci linked only with the intercept of SBP, suggesting that hypertension is heterogeneous and that genes for either the intercept or the slope also play roles in some cases of hypertension.

## Figures and Tables

**Table 1:** Correlation coefficients for SBP in males and females^A^

Sex	Age	21–30	31–40	41–50	51–60	>60
Male	10–20	0.507	0.483			
	21–30		0.606	0.422	0.242	0.436
	31–40			0.694	0.491	0.379
	41–50				0.692	0.537
	51–60					0.685
Female	10–20	0.532	0.420			
	21–30		0.567	0.474	0.632	0.542
	31–40			0.726	0.6002	0.489
	41–50				0.7632	0.645
	51–60					0.768

^A^*p *< 0.001 for all coefficients.
